# Gleanings from the German

**Published:** 1874-11-01

**Authors:** E. J. Doering


### GLEANINGS FROM THE GERMAN.

*Collated by Dr. E. J. Doering.*

### SULPHATE OF CADMIUM IN HAZINESS OF THE CORNEA.

DR. ANSIAUX uses the following mixture in all cases of haziness of the cornea with success, increasing the quantity of the cadmium as the eye bears it;

℞ Cadmii Sulphat. gr. j.

Muc. Gum Acaciæ,

Tinct. Opii, aa. f ʒ ij. M.

A few drops of this mixture are put into the eye by means of a camel's hair pencil twice or thrice daily, and the patient is directed to keep his eyes closed for ten minutes after each application of the remedy to prevent its being washed out with the tears. Although in this affection many opthalmologists use the tincture of opium alone, and therefore, would ascribe the good effect of the mixture to the opium contained in it, nevertheless experience has proven that tincture of opium alone does not cure such cases. Dr. A. therefore asserts positively that, in his opinion, sulphate of cadmium is a better remedy than opium, and that to it are due the good results which followed the use of the above mixture.

### GELSEMIUM SEMPERVIRENS.

Drs. Sayrer and Mackey, two Italian physicians, have been experimenting with gelsemium and write: "For several years this remedy has been used in America in the treatment of different forms of neuralgia and other nervous affections, the tincture being the form usually employed, in the dose of five to twenty drops. Dr. Ligg was the first to recommend its use in nervous toothache, and we have employed it with great success in all affections of the teeth, which were not complicated with inflammation of the gum or periosteum. We prescribe fifteen or twenty drops every six hours, and after the second or third dose the pain is gone. The remedy is especially valuable in allaying the irritability of the dental nerves in carious teeth and likewise in other forms of facial neuralgia. Some of the most obstinate cases of neuralgia and toothache which resisted all other medicinal agents, were cured rapidly by gelsemium. In large doses gelsemium causes poisonous symptoms, as disturbance of vision, diplopia, headache, and paralysis. Very small doses must be given to children to avoid all danger."

### INUNCTION OF CACAO BUTTER IN SCARLET FEVER.

Dr. Bayles writes as follows in the *Berl. Central Zeitung*: "Inunction of lard in scarlet fever, first recommended by Dr. Schneemann, has for years in Germany been used successfully to diminish the heat of the surface and to hasten desquamation. Instead of lard I greatly prefer cacao butter, as it is more cooling and refreshing to the patient, besides having a more agreeable odor. But aside from these properties I have found that it is readily absorbed by the skin and thus serves as a valuable nutritive agent. It is also more readily applied to the skin on account of its greater consistency than either lard or oils. If the fever is very high the inunction may be performed over parts of the body every hour, and occasionally the entire surface may be treated in this manner."

(As it has been proven that cacao butter is absorbed by the skin, and as it possesses nutritive properties besides its power of reducing the general temperature, and allaying pain and restlessness, it might be worth while to use these inunctions in inflammatory diseases, continued fevers, and especially in the profuse sweating of phthisis and rheumatism.)

### TREATMENT OF ECLAMPSIA.

In the *Berl. Beit. zur Geburtsk. und Gyœncok.*, Dr. Jaquet recommends the following treatment for uræmic eclampsia and eclampsia from acute anæmia of the brain, viz.: The patient must be completely enveloped in a large sheet dipped in water of 72° Fah., and well wrung out. Then cover the patient with a large woolen blanket, merely leaving the head uncovered, upon which an ice-bag is to be placed. If labor should be far advanced, the lower extremities must be wrapped up separately to avoid uncovering during the birth of the child. Ten minutes after the application of this envelopment the skin reddens, and in about an hour a free perspiration sets in, continuing as long as the sheet remains on. This treatment used during pregnancy is followed by no ill consequences, likewise, none need be feared after labor. After perspiration begins, the convulsions rapidly diminish, both in frequency and intensity, and the patient soon falls asleep. Chloroform, morphia, opium, or chloral hydrate may be used simultaneously. The patients never complain of a feeling of discomfort, even if the envelopments are continued for a longer time, nor was the life of the child ever endangered thereby.

